# Auditory Processing Intervention Program for school-aged children – development and content validation

**DOI:** 10.1590/2317-1782/20212021146en

**Published:** 2022-10-28

**Authors:** Cátia Luís, Ana Abrantes, Catarina Oliveira, Marisa Alves, Jorge Humberto Martins

**Affiliations:** 1 Escola Superior de Saúde da Universidade de Aveiro – ESSUA - Aveiro, Portugal.; 2 Centro Hospitalar e Universitário de Coimbra – CHUC - Coimbra, Portugal.

**Keywords:** Auditory Processing, Rehabilitation, Child, Portugal, Educational Status

## Abstract

**Purpose:**

The study aimed at the development and content validation of an Auditory Processing Intervention Program for school-aged European Portuguese speaking children with Auditory Processing Disorder.

**Methods:**

The first step was the program’s development and its instructions manual, which includes objectives, activities, procedures, materials, reinforcement, instructions, and verbal stimuli used, for the following auditory skills: auditory discrimination, auditory attention; auditory memory; auditory closure; figure-ground; auditory separation; auditory integration; binaural fusion; content validation was performed next, with two expert panels analyzing the program, through the use of a questionnaire. Content validity was calculated using the content validity index.

**Results:**

Program evaluation shows an excellent content validity. Some items were modified after analyzing the experts’ comments and suggestions (e.g. instructions, intensity differences, main character).

**Conclusion:**

This work allowed the development and content validation of an auditory processing intervention program, with verbal stimuli, selected according to strict linguistic criteria. In the future, the acceptability and efficacy of this program with the target population should be analyzed.

## INTRODUCTION

Auditory Processing Disorder (APD) is currently defined as a dysfunction of the central auditory system's ability to use the information sent by the peripheral auditory system^(1,2)^. It is expressed as difficulty in one or more auditory skills and culminates in an auditory information processing deficit, even with preserved peripheral hearing^(1-4)^. The auditory skills that integrate Auditory Processing (AP) are: sound localization and lateralization, auditory discrimination, recognition of auditory patterns, temporal auditory processing (temporal resolution, masking, integration, and ordering abilities), auditory performance with competitive acoustic signals (figure-ground), auditory performance in the presence of degraded acoustic signals (closure) and binaural fusion (dichotic listening – binaural separation and integration –, binaural interaction and interhemispheric integration)^(5)^.

APD has a multifactorial etiology and may result from neuroanatomical abnormalities, such as a delay in the maturation of the central nervous system or exposure to exogenous factors (e.g. tobacco, alcohol) during the critical periods of brain development^(6,7)^.

Individuals with APD generally present difficulties with language, learning, understanding verbal instructions, especially when the input is presented at a fast speech rate, with auditory discrimination of minimal pairs, identification of people's voices, sound localization, and musical or singing skills^(7-12)^. APD can also impair the children's social performance, for instance, by restricting classroom activities and participation^(11)^.

It is estimated that 2% to 5% of the school-aged population suffers from APD^(13)^. However, it affects about 30% to 50% of children with learning disabilities^(14)^, as well as about 52% of children with dyslexia and/or developmental language disorders^(15)^.

APD intervention includes *bottom-up* (acoustic signal improvement and auditory training) in addition to *top-down* (cognitive, linguistic, and metacognitive strategies) approaches. It should be planned by a multidisciplinary team that integrates speech-language pathologists (SLPs) and audiologists, and may also include psychologists, teachers and occupational therapists^(6,10)^. This intervention should be implemented as early as possible, it requires intensive auditory training and must be consistent with the previous diagnosis, to develop the neuroplasticity that characterizes the auditory nervous system^(2,3,8)^.

Such treatment may undergo *environmental modifications, compensatory strategies* (cognitive-linguistic skills training), or *direct remediation measures* ^(2,3,10,16)^. Environmental modifications and compensatory strategies aim at reducing the impact of APD on individuals' daily lives, and direct remediation (auditory training) aims at reducing AP alterations^(4)^.

Auditory training programs encompass activities that focus on the identified skills deficits^(9,17)^. These activities should include varied tasks; with comfortable stimulus intensity; they should be presented systematically and in increasing degrees of difficulty, to provide variation and motivation, with *feedback* and positive reinforcement; they must accommodate the differences between ears (left and right), advancing only when adequate performance is obtained for both ears, and should promote the intensive practice, preferably in a daily basis, during the established intervention period^(8,9)^.

Although the auditory training duration is not a consensus in the literature, twenty to thirty minutes of practice is usually recommended, from three to four times a week, for at least six weeks, varying according to the number of affected skills^(17)^. As for the difficulty level of the auditory training, a performance below 30% indicates that the task is too demanding. On the other hand, to achieve progress in the auditory training, the patient's success rate should be between 70% and 80%^(3,17,18)^.

Auditory training is effective in the rehabilitation of auditory skills, improving the perception of more complex acoustic signals, such as speech^(10,17,19,20)^. Furthermore, when including activities that target temporal processing skills, also improves the children's reading performance^(21)^.

In recent years, several intervention programs have been developed to contemplate speech sounds and nonverbal vocalization and stimulate different auditory skills, combined with language and memory tasks (e.g.: Afinando o Cérebro, Active Listening, LiSN&Learn, Fast ForWord)^(20,22-24)^.

Many of these programs, adapted for tablets and smartphones (CBAT – computer-based auditory training)^(12)^, display a pleasant aesthetic format, with multisensory stimulation, *feedback*, positive reinforcement, and opportunity for intensive and adaptive training, thus becoming an effective tool, especially for the pediatric population presenting speech disorders, learning disabilities and reading difficulties concomitant with AP alterations^(8)^.

Nevertheless, in the case of European Portuguese (EP) speakers, there is no validated APD intervention program whose effectiveness has been actually assessed. That being said, in the case of nonverbal vocalizations, it is possible to use the programs available in other languages, while regarding verbal sounds, these programs are not directly functional for the population whose first language is EP, since the auditory training must occur in the patient's language^(8)^.

Considering the scarcity of structured and validated programs for APD intervention with children, which constrains the SLPs' evidence-based practice, the present study's purpose was to develop and validate an AP intervention program for school-aged children (from six to ten years old), for EP speakers, which contemplated activities that stimulate auditory skills that are more dependent on verbal stimuli.

## METHODS

A cross-sectional exploratory and descriptive study were conducted with a quantitative approach, and content validation was performed with a Panel of Experts (PE)^(25)^. Since the study does not involve direct participation of human beings, it was not considered necessary to apply for ethics committee approval, nor was there a need for drafting informed consent forms.

### Development of the Auditory Processing Intervention Program

The present intervention program aims to stimulate auditory skills related to auditory discrimination, auditory attention; auditory memory; auditory closure; figure-ground; auditory separation; auditory integration, and binaural fusion. PIPA (an acronym for “Auditory Processing Intervention Program” in Portuguese, or “Programa de Intervenção em Processamento Auditivo”) comes with a playful activities framework that displays motivating scenarios and a reward system. The activities are hierarchized according to their difficulty degree, they are intended to meet specific objectives for the stimulation and to train each one of the targeted auditory skills.

The verbal stimuli included in each activity were carefully chosen, based on strict linguistic criteria, namely extension and syllabic structure of the word. Thus, for all the PIPA activities, the stimuli contemplate monosyllabic, disyllabic, trisyllabic, and polysyllabic words in a percentage similar to the frequency of the occurrence in EP^(26)^. As for the syllabic structure, stimuli with every possible syllabic format in EP were selected, respecting their frequency of occurrence^(27,28)^. It was not possible to meet these linguistic criteria only in cases where the stimuli pertained to specific semantic fields.

Each section was organized by levels, in an ascending order of difficulty, and all activities must be completed individually, monitored by an SLP. Both the child and the SLP must use headphones, not requiring an acoustic booth. In each game/task, about 10 to 15 consecutive stimuli are presented and, if the child scores 75% of correct answers, he/she can level up.

In some games, the SLP may manipulate the conditions, such as the stimuli intensity variation, the signal-to-noise ratio, the temporal variation of the stimuli presentation in dichotic listening, and the selection of the ear for stimuli presentation (right ear vs. left ear). Additionally, it is possible to monitor the child's performance/progress.

PIPA also comes with a manual, which includes the program's objectives and respective tasks, the framework, the task's description/procedures, the instructions, the provided *feedback*/reward, the materials used, and the stimuli involved. Even though the program includes activities to stimulate various auditory skills, each child will only explore the spaces that the SLP determines, according to the established intervention plan, necessarily following an evaluation previously performed by an audiologist^(1,4,26)^. The SLP is free to choose whether to start with the stimuli in the right ear or the left ear, and the child will have to perform the tasks in both ears to level up.

**Figure 1:**
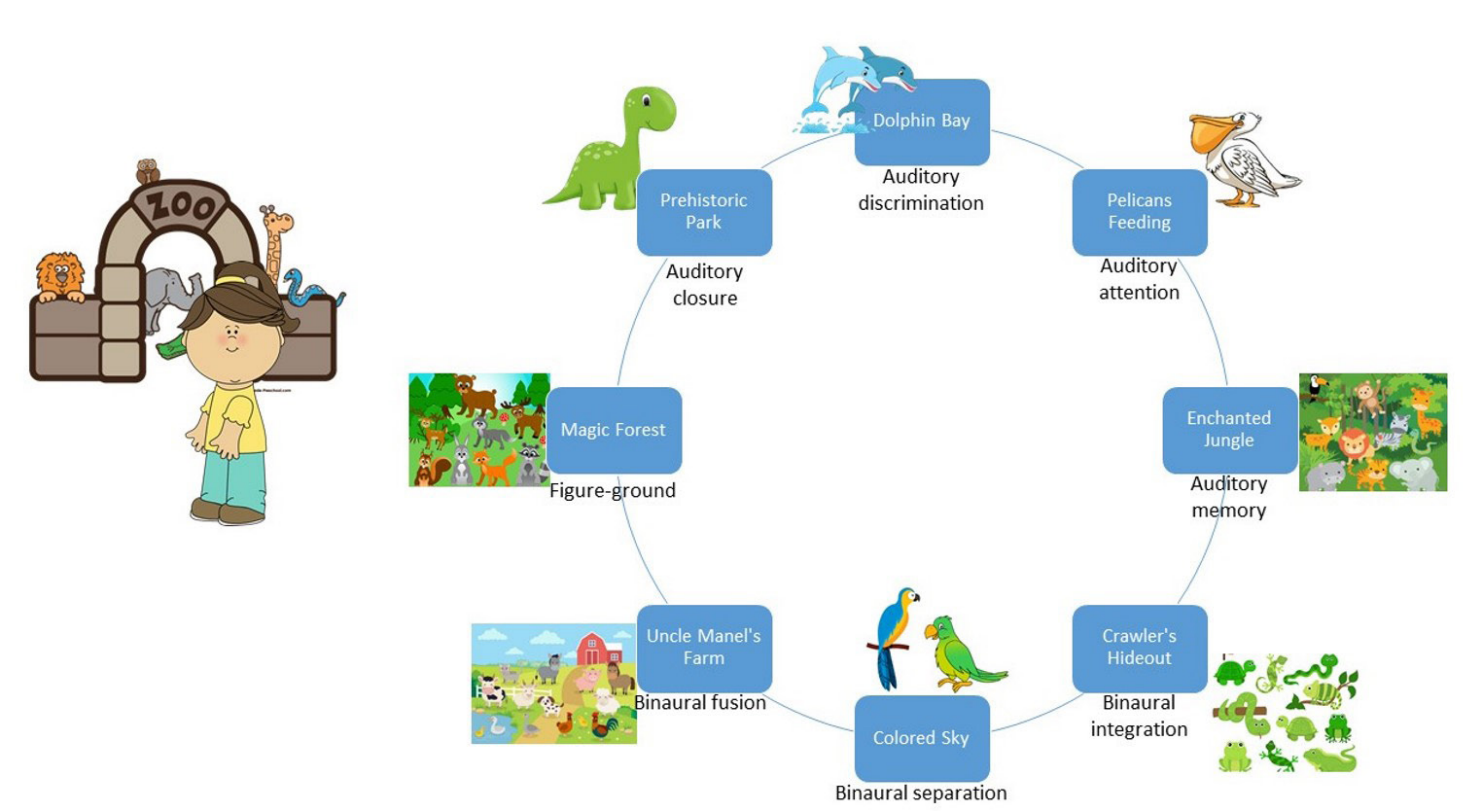
Stimulated skills in each space of PIPA's zoo

PIPA's framework revolves around the story of a girl who visits a zoo, with several spaces/habitats where she can conquer the animals that are there. To do this, she has to perform the tasks that stimulate different auditory skills (*dolphin bay* – auditory discrimination; *pelican feeding* – auditory attention; *enchanted jungle* – auditory memory; *vibrant sky* – binaural separation; *crawlers' nest* – binaural integration; *Mr. Manel's farm* – binaural fusion; *prehistoric park* – closure; *enchanted forest* – figure-ground). Each section of PIPA aims to train a basic auditory skill, and, of course, other abilities will be stimulated further on^(26)^. Figure 1 presents a schematic view of the program.

In Table 1, the spaces outlined within PIPA are described, as well as the targeted skills, the number of levels, the activities, the type of verbal stimuli used, and the reinforcement that is given to the child.

**Table 1:** PIPA's framework

**Space**	**Targeted Skills**	**No. of Levels**	**Name of the Activity**	**Type of Stimuli**
Dolphin Bay	Auditory discrimination	4	The Shark Scare	Consonant-Vowel Syllables
			The Dolphin's Jump	Words
			Soraia, the Ray	Words
			The Seahorse's Kiss	Pseudowords
Pelican Feeding	Auditory Attention	3	Tadpoles	History
			Birds	History
			Insects	Music
Enchanted Forest	Auditory Memory	5	The Rhino's Horn	Words of the semantic category related to colors
			The Lion's Mane	Words from the semantic category related to food
			The Zebra's Stripes	Words
			The Elephant's Trunk	Pseudowords
			The Giraffe's Neck	History
Prehistoric Park	Closure	4	Pterodactylus	Sets of three equal/one different words+ noise
			Diplodocus	Sets of three equal/one different words+ noise
			Triceratops	Simple sentences + noise
			T-Rex	Complex phrases + noise
Vibrant Sky	Binaural separation	5	Hunter Owl	Words
			Talking Parrot	Pairs of similar words
			How many babies can the stork carry?	Pairs of similar words
			The Pigeon Postman	Words (presented two at a time in each ear)
			The flight of the eagle	Simple sentences
Crawlers' Nest	Binaural integration	4	Snake Labyrinth	Words
			Iguana's log	Words
			How many colors does the chameleon have?	Simple sentences
			How many worms does the salamander eat?	Sentences with ungrammatical semantic elements.
Mr. Manel's farm	Binaural fusion	4	The laying hen	Phrases
			The glutton rabbit	Words
			The hungry pig	Pseudowords
			The jumping goat	History and questions
The Enchanted Forest	Figure-ground	3	The squirrel	History and words
			The wolf	Complex sentences
			The fox	Simple sentences

### Content validation

PIPA's research validity was performed through content validation, which verifies if the instrument is compatible with its proposition, indicating that the program’s design was planned following a careful conceptual analysis, both the assessment and evaluation of the content relevance were carried out by a group of experts^(25)^.

One way to assess the validity of the content is through the Content Validation Index (CVI)^(29)^. This method uses a 4-point ordinal scale, the lowest ranking response being “strongly disagree/not relevant” and the highest being “strongly agree/highly relevant”^(29)^. The CVI is determined by calculating the number of items graded with 3 or 4, divided by the total number of items^(29)^. A value of 0.78 was used as a reference to determine the content validity^(29)^.

To validate PIPA's content, regarding the contents' scope, intelligibility, adequacy, and relevance, two panels of experts were constituted, based on the criteria outlined in the literature^(25)^, namely, clinical experience in the AP field (minimum of 5 years) and theoretical knowledge in the area of study. The decision to select two different panels of experts was because a single PE was considered to be insufficient to assess such a wide range of tasks, associated with a broad amount of different stimuli.

Experts were selected according to the non-probability convenience sampling method. The first panel consisted of five experts who analyzed the tasks and the manual of auditory skills related to auditory discrimination, auditory attention, auditory memory, and closure, and the second panel consisted of six experts who analyzed skills related to binaural separation, binaural integration, binaural fusion, and figure-ground.

PIPA's manual was sent after the first contact by e-mail, requesting the collaboration of the experts in the study. The professionals were asked to complete a questionnaire divided into two parts: sociodemographic characterization and PIPA's content analysis (fourteen statements, ranking from one to four)^(25,29)^. With this questionnaire, it was intended to attest to the fulfillment of the inclusion criteria to integrate the PE, and also to evaluate the opinion of the experts regarding the following items: program utility, suitability to the clinical practice and the target audience, selected auditory skills, instructions, framework, rewards, tasks, stimulus (quantity and selection) and organization.

## RESULTS

The sociodemographic characterization of the experts who analyzed PIPA is described in Table 2.

**Tabela 2:** Constitution of the PE

**Subjects Identification**		**Gender**	**Educational Stage**	**Years of Profession Activity**	Intervention*	**Degree** *	Instructor*
**PE1**	Subject 1	Female	Licentiate	5	Yes	Yes	No
	Subject 2	Female	Licentiate	27	Yes	Yes	No
	Subject 3	Female	Doctorate	17	No	Yes	No
	Subject 4	Female	Doctorate	17	Yes	Yes	Yes
	Subject 5	Female	Licentiate	20	Yes	Yes	No
**PE2**	Subject 1	Female	Licentiate	14	Yes	Yes	Yes
	Subject 2	Female	Licentiate	20	Yes	Yes	Yes
	Subject 3	Female	Licentiate	19	Yes	Yes	Yes
	Subject 4	Female	Master's Degree	8	Yes	Yes	No
	Subject 5	Female	Master's Degree	14	Yes	Yes	No
	Subject 6	Female	Doctorate	12	Yes	Yes	Yes

* Regarding the AP scope

All experts met the pre-defined criteria of clinical experience and specific knowledge in the AP field, highlighting the fact that out of the eleven experts, five had previous experience with AP training. At the time of the study, one of the experts (Subject 3 – PE 1) held a teaching position, and also had previous experience with APD intervention.

The overall CVI obtained with PIPA's validation was 0.95. The quantitative results obtained by PE 1 and PE 2 are presented in Table 3 and Table 4, respectively.

**Table 3:** Consensus between the PE1 members, regarding PIPA

**Items to validate/validated**	**CVI**
1. These materials are useful for clinical practice.	1
2. The selected auditory skills are adequate.	1
3. The instructions are clear and have practical relevance	0.8
4. The program is suitable for school-aged children with Auditory Processing Disorder.	1
5. The program's framework (a girl visiting a zoo) is appropriate.	1
6. The division of spaces to visit in the zoo in connection to the selected skills is appropriate.	1
7. The *feedback* reward given in each activity is adequate.	1
8. The tasks included in the *auditory discrimination section (dolphin bay)* allow for the appropriate intervention with children with difficulties related to said skill	1
9. The tasks included in the *auditory attention section (pelicans' feeding)* allow for the appropriate intervention with children with difficulties related to said skill.	1
10. The tasks included in the *auditory memory section (enchanted forest*) allow for adequate intervention with children with difficulties related to said skill.	1
11. The tasks included in the *closure section (prehistoric park)* allow for adequate intervention with children with difficulties related to said skill.	0.6
12. The stimuli selected for each of the tasks are adequate.	0.8
13. The number of stimuli included in the tasks is adequate.	1
14. Within each skill, the organization of tasks at difficulty levels is adequate.	1
**PE1 overall total**	**0.94**

**Table 4:** Consensus between the PE2 members, regarding PIPA

**Items to validate/validated**	**CVI**
15. These materials are useful for clinical practice.	1
16. The selected auditory skills are adequate.	1
17. The instructions are clear and have practical relevance.	0.83
18. The program is suitable for school-aged children with Auditory Processing Disorder.	1
19. The program's framework (a girl visiting a zoo) is appropriate.	0.83
20. The division of spaces to visit in the zoo in connection to the selected skills is appropriate.	1
21. The *feedback* reward given in each activity is adequate.	1
22. The tasks included in the binaural *separation section (Vibrant Sky*) allow for the appropriate intervention with children with difficulties related to said skill.	1
23. The tasks included in the *binaural integration section (Crawlers' Nest)* allow for the appropriate intervention with children with difficulties related to said skill.	0.83
24. The tasks included in the *binaural fusion section (Mr. Manel's Farm)* allow for the appropriate intervention with children with difficulties related to said skill.	1
25. The tasks included in the *figure-ground section (Enchanted Forest)* allow for the appropriate intervention with children with difficulties related to said skill.	1
26. The stimuli selected for each of the tasks are adequate.	1
27. The stimuli selected for each of the tasks are adequate.	1
28. Within each skill, the organization of tasks at difficulty levels is adequate.	1
**PE2 overall total**	**0.96**

Although it was not necessary to completely reformulate any item, since the content of all items was validated, a few modifications were made to PIPA to adhere to some of the experts' suggestions, documented in the observations/suggestions section.

As for the program's framework, at the suggestion of the experts, we introduced the possibility for the child to choose the gender (male/female) of PIPA's main character.

Changes were made to the tasks' instructions of the following spaces: *Vibrant Sky, Crawlers' Nest, Mr. Manel's Farm,* and *Enchanted Forest,* striving to provide the SLP and the child with a better understanding of the activities, using shorter sentences and giving examples. Training components were added to all tasks, at the suggestion of the experts, to facilitate the understanding of the activities.

In addition, three pairs of stimuli were altered in the task *The Dolphin Jump*, from the *Dolphin Bay* space, to increase the percentage of dissyllabic words with phonemes in the word-medial position. The number of stimuli was increased in the task *The Seahorse's Kiss*, in the *Dolphin Bay* space, going from five to ten pairs of pseudowords, according to the recommendation of the experts. Still regarding the stimuli, at the suggestion of the experts, some sentences were altered in the activities *The Eagle's Flight*, from the *Vibrant Sky*space (binaural separation), and *How Many Colors has the Chameleon?*, from the *Crawlers' Nest* (binaural integration) to standardize its grammatical structure.

It was also accepted the suggestion of not inserting phrases with ungrammatical semantic and syntactic elements in the same group of stimulus sentences, in the task *How Many Worms does the Salamander Eats?* from the *Crawlers' Nest* space (binaural integration), opting for using phrases with only ungrammatical semantic elements. The lexicon of some phrases was also reviewed, on the grounds of being associated with a particular dialect, on the risk of not being familiar with children from other geographical locations.

In the binaural separation tasks (*Vibrant Sky*), the introduction of a greater intensity variation (20 dB, 15 dB, 10dB, 5dB, and without variation) was contemplated between the stimuli that come through the right side vs. left side, similarly, we accepted the suggestion of introducing the possibility of manipulating the intensity variation (20 dB, 15 dB, 10dB, 5dB and without variation) in the tasks related to figure-ground (*Enchanted Forest*), to assure PIPA's applicability in the cases of children with a more severe disorder and/or with associated hearing loss issues.

The task-related to auditory memory of colors (level 1 – *The Rhino's Horn*, in *The Enchanted Forest* space) was also reformulated, given that one of the experts pointed out potential color blindness complications. In these situations, the task can be performed with the support of the SLP, who can select the colors after the child indicates the sequence of colors they heard.

In the case of item 11 of PE1, there was no need for alterations, even after presenting a 0.6 CVI score, given that, upon completing the questionnaire, some of the PE members did not understand that it was a task in which white noise and/or distortion were used.

These amendments are summarised in Table 5.

**Table 5:** Amendments made to PIPA, at the suggestion of the experts

**Space**	**Alteration**	
Program framework	Gender choice (male/female) of PIPA's main character	
Vibrant Sky	Alteration in instructions	Introduction of a higher intensity variation (20dB, 15dB, 10dB, 5dB and without variation) between the stimuli
Crawlers' Nest		
Mr. Manel's Farm		
The Enchanted Forest		Intensity variation manipulation (20dB, 15dB, 10dB, 5dB and no variation)
The Dolphin's Jump	Alteration of three pairs of stimuli	
The Seahorse's Kiss	Increased number of stimuli	
The flight of the Eagle	Alteration of some sentences	
How many colors does the chameleon have?		
How many worms does the salamander eat?	Conservation of sentences with only ungrammatical semantic elements. Review of the entire lexicon	
The Rhino's Horn	Reformulation of the task to allow it to be performed by children with color blindness.	
All Activities	Introducing training items	

## DISCUSSION

The selection of two panels of experts, one with five members and the other with six, proved to be appropriate since it complied with the defined range of five to ten experts established by the literature^(25)^. The fact that the experts had clinical experience and training in the AP field evidence that the evaluator is familiar with this area of work, which justifies, from the outset, their inclusion in the PE.

Although the members of each PE did not fully know PIPA, hindering the requested analysis work, the requirements of a content validation study were fulfilled, selecting specialists from various geographical areas of the continental national territory that met all the inclusion criteria^(25)^.

The overall CVI obtained (0.95) amounts to an excellent content validity ranking, since it is greater than 0.90^(25,29,30)^. However, amendments were made as there was a consensus among experts concerning the suggested alterations^(29)^. Under other conditions, Polit & Beck^(29)^ would argue that the literature should be reviewed once more to improve the program.

The development and validation of an intervention program, particularly with regard to the AP field, is an innovative factor for EP. In this context, and given the scarcity of materials, it was decided to create a program in which verbal stimulus is used for the training of different auditory skills^(10,17)^. In this sense, it was essential to carefully select PIPA's verbal stimuli, balancing the input according to the frequency patterns occurring in EP^(27,28)^.

For PIPA's elaboration, a careful selection of skills, objectives, tasks, and stimuli was undertaken, always being mindful of its use in the clinical context. At the same time, the fact that the program targets school-aged children were kept in mind, and, as such, the use of fun tools was considered necessary as a playful way of motivating and engaging kids^(8,9)^.

The inclusion of a system of rewards and monitoring of the children's correct answers, aside from the experts' unanimous validation, was deemed a fundamental factor for the child's continuous evaluation, for the (re)definition of intervention goals, and the maintenance of the task's motivational indexes, following what is endorsed in the literature for intervention in APD cases^(9,18)^. Moreover, it is in line with some programs available internationally, which show the child's progress with the auditory training activities^(20,22)^.

The auditory skills developed with PIPA (auditory discrimination, auditory attention, auditory memory, binaural separation, binaural integration, binaural fusion, closure, and figure-ground) allow for speech understanding since they demand discrimination, recognition, selective and sustained attention, as well as the ability to memorize sounds^(11)^. Hence, given that APD can have negative consequences on the individuals' linguistic, social and academic performance^(7,11)^, PIPA can have a positive impact on its users, concerning the personal factors that promote activity and participation in multiple contexts.

In addition, evidence-based practice in decision making is essential to raise the quality of the therapeutic intervention^(7)^. PIPA thus paves the way for other studies in the context of APD intervention for children whose first language is EP, contributing to the improvement of the SLP's clinical practice in this field. As a future endeavor, studies must be conducted on PIPA's acceptability and efficacy for children with and without APD.

## CONCLUSION

This research allowed for PIPA's development and validation, meeting the steps defined in the literature for the creation of new instruments. This is an innovative instrument for EP speakers, with an excellent CVI, with an acceptability and effectiveness analysis foreseen in future studies.
