# Supplementary material for: Auditory Processing Intervention Program for school-aged children – development and content validation
Source: Codas. 2022 Oct 28;35(1):e20210146. doi: 10.1590/2317-1782/20212021146en (PMC10010500; doi:10.1590/2317-1782/20212021146en)
Supplement: Translation

# Translation: Desenvolvimento e validação de conteúdo de um Programa de Intervenção em Processamento Auditivo para crianças em idade escolar

**DOI:** 10.1590/2317-1782/20212021146pt

**Authors:** Cátia Luís, Ana Abrantes, Catarina Oliveira, Marisa Alves, Jorge Humberto Martins

**Affiliations:** 1 Escola Superior de Saúde da Universidade de Aveiro – ESSUA - Aveiro, Portugal.; 2 Centro Hospitalar e Universitário de Coimbra – CHUC - Coimbra, Portugal.

**Keywords:** Processamento Auditivo, Reabilitação, Criança, Portugal, Escolaridade

## Abstract

**Objetivo:**

O estudo teve como objetivos o desenvolvimento e validação de conteúdo de um Programa de Intervenção em Processamento Auditivo, destinado a crianças em idade escolar, com perturbação do processamento auditivo, falantes do português europeu.

**Método:**

A primeira etapa consistiu no desenvolvimento do programa e respetivo manual de instruções, que inclui objetivos, atividades, procedimentos, materiais, reforços, instruções e estímulos verbais utilizados, para as competências auditivas de discriminação auditiva, atenção auditiva, memória auditiva, fechamento, figura-fundo; separação binaural, integração binaural e fusão binaural; a segunda etapa consistiu na validação de conteúdo, por dois painéis de peritos que analisaram o programa, através de um questionário. A validade de conteúdo foi calculada usando o índice de validade de conteúdo.

**Resultados:**

A avaliação do programa revela uma excelente validade de conteúdo. Alguns itens foram modificados depois da análise dos comentários e sugestões dos peritos (ex: instruções, desníveis de intensidade, personagem principal).

**Conclusão:**

O estudo permitiu o desenvolvimento e validação de um programa de intervenção em processamento auditivo, com estímulos verbais, selecionados de acordo com critérios linguísticos rigorosos. Futuramente, está prevista a realização de estudos de aceitabilidade e eficácia do programa junto da população alvo.

## INTRODUÇÃO

A Perturbação do Processamento Auditivo (PPA) é atualmente definida como um défice na capacidade do sistema auditivo central para usar as informações enviadas pelo sistema auditivo periférico^(1,2)^. Traduz-se por uma dificuldade numa ou mais competências auditivas e culmina num défice do processamento da informação auditiva, mesmo com a audição periférica preservada^(1-4)^. As competências auditivas que integram o Processamento Auditivo (PA) são: a localização e lateralização sonora, a discriminação auditiva, o reconhecimento de padrões auditivos, o processamento auditivo temporal (resolução, mascaramento, integração e ordenação temporais), o desempenho auditivo com sinais acústicos competitivos (figura-fundo), o desempenho auditivo na presença de sinais acústicos degradados (fechamento) e o processamento binaural (escuta dicótica – separação binaural e integração binaural –, interação binaural e integração inter-hemisférica)^(5)^.

A PPA apresenta etiologia multifatorial, podendo ser resultado de uma anomalia neuroanatómica, de um atraso na maturação do sistema nervoso central ou da exposição a fatores exógenos (ex: tabaco, álcool) durante períodos críticos do desenvolvimento cerebral^(6,7)^.

Os indivíduos com PPA apresentam, geralmente, dificuldades na linguagem, na aprendizagem, na compreensão de instruções orais, sobretudo quando estas são apresentadas em velocidade de fala rápida, na discriminação de palavras com pares mínimos, na identificação das vozes das pessoas, na localização dos sons e nas competências musicais ou de canto^(7-12)^. A PPA pode prejudicar também o desempenho social das crianças, restringindo, por exemplo a atividade e participação na sala de aula^(11)^.

Estima-se que a PPA afete cerca de 2% a 5% da população em idade escolar^(13)^. Contudo, atinge cerca de 30% a 50% das crianças com dificuldades de aprendizagem^(14)^, bem como cerca de 52% das crianças com dislexia e/ou perturbação do desenvolvimento da linguagem^(15)^.

A intervenção na PPA inclui abordagens *bottom-up* (melhoria do sinal acústico e treino auditivo) e *top-down* (estratégias cognitivas, linguísticas e metacognitivas) e deve ser planeada no seio de uma equipa multidisciplinar que integre terapeutas da fala (TFs) e audiologistas, podendo também incluir psicólogos, professores e terapeutas ocupacionais^(6,10)^. Esta intervenção deve ser implementada o mais precocemente possível, deve incluir treino intensivo e deve estar de acordo com o diagnóstico previamente realizado, de modo a explorar a plasticidade neuronal que caracteriza o sistema nervoso auditivo^(2,3,8)^.

A intervenção pode passar por *modificações ambientais*, *estratégias compensatórias* (treino de competências cognitivo-linguísticas) ou *remediação direta* ^(2,3,10,16)^. As modificações ambientais e as estratégias compensatórias visam a diminuição do impacto da PPA no dia-a-dia dos indivíduos e a remediação direta (treino auditivo) visa a redução do défice no PA^(4)^.

Os programas de treino auditivo incluem atividades focadas nas competências identificadas como deficitárias^(9,17)^. Estas atividades devem incluir tarefas variadas; estímulos com níveis de intensidade confortáveis; devem ser apresentadas sistematicamente e em graus de dificuldade crescente, de modo a promover a mudança e a motivação, com *feedback* e reforço positivo; devem atender às diferenças entre ouvidos (esquerdo e direito), avançando somente quando se obtém um desempenho adequado para os dois ouvidos e devem promover a prática intensiva, se possível diária, durante o tempo estabelecido para a intervenção^(8,9)^.

A duração do treino auditivo não está bem estabelecida na literatura, porém é comum a indicação de que o mesmo deverá ocorrer durante vinte a trinta minutos, entre três a quatro vezes por semana, durante pelo menos seis semanas, variando consoante o número de competências alteradas^(17)^. No que respeita à dificuldade do treino auditivo, um desempenho abaixo dos 30% indica que a tarefa é demasiado exigente. Por outro lado, para que o paciente possa avançar no treino auditivo, a taxa de acerto deverá situar-se entre os 70% e os 80%^(3,17,18)^.

O treino auditivo tem-se mostrado eficaz na reabilitação das competências auditivas, melhorando a perceção de sinais acústicos mais complexos, como por exemplo, a fala^(10,17,19,20)^. Para além disso, quando inclui atividades direcionadas para as competências de processamento temporal, também melhora o desempenho das crianças ao nível da leitura^(21)^.

Nos últimos anos, têm sido desenvolvidos vários programas de intervenção que contemplam sons verbais e não-verbais e estimulam diferentes competências auditivas, combinadas com algumas tarefas de linguagem e memória (ex.: Afinando o Cérebro, Escuta Ativa, LiSN&Learn, Fast ForWord)^(20,22-24)^.

Muitos destes programas, adaptados para *tablets* e *smartphones* (CBAT – computer-based auditory training)^(12)^, apresentam um formato estético agradável, com estimulação multissensorial, *feedback*, reforço positivo e oportunidade para treino intensivo e adaptativo, tornando-se assim numa ferramenta eficaz, sobretudo para a população pediátrica que apresenta dificuldades de linguagem, de aprendizagem e de leitura concomitantes com as alterações no PA^(8)^.

No entanto, para o Português Europeu (PE) não se conhece nenhum programa de intervenção em PPA validado e cuja eficácia tenha sido aferida. Ainda que, no caso dos sons não verbais, seja possível recorrer aos programas disponíveis noutras línguas, no que respeita aos sons verbais, estes programas não são diretamente utilizáveis pela população cuja língua materna é o PE, uma vez que o treino auditivo deverá ocorrer na língua do paciente^(8)^.

Tendo em conta a escassez de programas estruturados e validados para intervenção com crianças com PPA, o que condiciona a prática baseada na evidência por parte dos TFs, este estudo teve como propósito o desenvolvimento e validação de um programa de intervenção em PA para crianças em idade escolar (dos seis aos dez anos), falantes do PE, que incluísse atividades para estimular as competências auditivas mais dependentes dos estímulos verbais.

## MÉTODO

O estudo desenvolvido é exploratório, transversal e descritivo com uma abordagem quantitativa, sendo a validação de conteúdo realizada através de um Painel de Peritos (PP)^(25)^. Dado que o estudo não envolve diretamente a participação de seres humanos, não se considerou necessário proceder a qualquer pedido de parecer à comissão de ética, nem houve lugar a um termo de consentimento livre e esclarecido.

### Desenvolvimento do Programa de Intervenção em Processamento Auditivo

Este programa de intervenção visa estimular as competências auditivas de discriminação auditiva, atenção auditiva, memória auditiva, fechamento, figura-fundo, separação binaural, integração binaural e fusão binaural. O PIPA inclui atividades lúdicas, com cenários motivadores e um sistema de recompensas. As atividades estão hierarquizadas em função do grau de dificuldade e cumprem objetivos específicos para a estimulação e treino de cada uma das competências auditivas selecionadas.

Os estímulos verbais incluídos em cada uma das atividades foram selecionados cuidadosamente, tendo por base critérios linguísticos rigorosos, nomeadamente extensão e estrutura silábica da palavra. Desta forma, para cada uma das atividades do PIPA, os estímulos contemplam palavras monossilábicas, dissilábicas, trissilábicas e polissilábicas numa percentagem próxima da frequência do PE^(26)^. Quanto à estrutura silábica, selecionaram-se estímulos com todos os formatos silábicos possíveis no PE, respeitando a sua frequência de ocorrência^(27,28)^. Apenas nos casos em que os estímulos dizem respeito a campos semânticos específicos, não foi possível atender a estes critérios linguísticos.

Cada secção está organizada por níveis, por ordem crescente de dificuldade, sendo que todas as atividades devem ser realizadas individualmente, com acompanhamento de um TF. Tanto a criança como o TF devem usar auscultadores, não sendo necessária uma cabine acústica. Em cada jogo/tarefa são apresentados cerca de 10 a 15 estímulos consecutivos e, se a criança atingir 75% de acertos, pode avançar de nível.

Em alguns jogos, o TF poderá manipular as condições, tais como desníveis de intensidade dos estímulos, relação sinal-ruído, desnível temporal de apresentação dos estímulos em escuta dicótica e seleção do ouvido de apresentação dos estímulos (ouvido direito *vs.* ouvido esquerdo). Poderá ainda monitorizar o desempenho/progresso da criança.

O PIPA inclui também um manual, que contempla os objetivos do programa e as respetivas tarefas, o enquadramento, a descrição/procedimentos das tarefas, as instruções, o *feedback*/recompensa fornecidos, os materiais utilizados e os estímulos envolvidos. Apesar do programa incluir atividades de estimulação de várias competências auditivas, cada criança apenas visitará os espaços que o TF definir, de acordo com o plano de intervenção traçado, implicando obrigatoriamente uma avaliação realizada previamente por um audiologista^(1,4,26)^. O TF tem a possibilidade de escolher se quer iniciar com os estímulos no ouvido direito ou no ouvido esquerdo, sendo que a criança terá sempre que realizar as tarefas nos dois ouvidos para poder passar de nível.

O enquadramento do PIPA consiste numa menina que visita um jardim zoológico, com vários espaços/habitats onde pode conquistar os animais que lá se encontram. Para tal, terá de executar as tarefas que estimulam as diferentes competências auditivas (*baía dos golfinhos* – discriminação auditiva; *alimentação dos pelicanos* – atenção auditiva; *selva encantada* – memória auditiva; *céu colorido* – separação binaural; *esconderijo dos rastejantes* – integração binaural; *a quinta do tio Manel* – fusão binaural; *parque pré-histórico* – fechamento; *floresta mágica* – figura-fundo). Cada secção do PIPA tem como objetivo o treino de uma competência auditiva principal, sendo que, naturalmente, haverá outras competências estimuladas^(26)^. A Figura 1 apresenta um esquema do programa.

**Figura 1:** Competências estimuladas em cada espaço do jardim zoológico do PIPA

Na Tabela 1, encontram-se descritos os espaços contemplados no PIPA, bem como as competências estimuladas, o número de níveis, as atividades, o tipo de estímulos verbais utilizados e o reforço que é dado à criança.

**Tabela 1:** Constituição do PIPA

**Espaço**	**Competência estimulada**	**Nº de níveis**	**Nome da atividade**	**Tipo de estímulos**
Baía dos Golfinhos	Discriminação auditiva	4	O susto do tubarão	Sílabas Consoante-Vogal
			O salto do Golfinho	Palavras
			Soraia, a raia	Palavras
			O beijinho do cavalo marinho	Pseudopalavras
Alimentação dos Pelicanos	Atenção auditiva	3	Girinos	História
			Aves	História
			Insetos	Música
Selva Encantada	Memória auditiva	5	O corno do rinoceronte	Palavras da categoria semântica cores
			A juba do leão	Palavras da categoria semântica alimentos
			As riscas da zebra	Palavras
			A tromba do elefante	Pseudopalavras
			O pescoço da girafa	História
Parque Pré-histórico	Fechamento	4	Pterodáctilo	Conjuntos de três palavras iguais/uma diferente + ruído
			Diplodocos	Conjuntos de três palavras iguais/ uma diferente + ruído
			Triceratops	Frases simples + ruído
			T-Rex	Frases complexas + ruído
Céu colorido	Separação binaural	5	Coruja caçadora	Palavras
			Papagaio falante	Pares de palavras parecidas
			Quantos bebés transporta a cegonha?	Pares de palavras parecidas
			O carteiro pombo	Palavras (apresentadas duas de cada vez em cada ouvido)
			O voo da águia	Frases simples
Esconderijo dos rastejantes	Integração binaural	4	Labirinto da cobra	Palavras
			Tronco da iguana	Palavras
			Quantas cores tem o camaleão?	Frases simples
			Quantas minhocas come a salamandra?	Frases com agramaticalidade semântica.
A quinta do tio Manel	Fusão binaural	4	Galinha poedeira	Frases
			Coelho comilão	Palavras
			O porco esfomeado	Pseudopalavras
			Cabra saltitona	História e perguntas sobre a mesma
Floresta Mágica	Figura-fundo	3	Esquilo	História e palavras
			Lobo	Frases complexas
			Raposa	Frases simples

### Validação de conteúdo

A validação do PIPA foi realizada através da validação de conteúdo, que verifica se o instrumento corresponde exatamente ao que se propõe, implicando a conceção do mesmo de acordo com uma análise conceptual cuidada e a análise e avaliação da relevância desse conteúdo por um grupo de especialistas^(25)^.

Uma das formas de aferir a validade de conteúdo é através do Índice de Validade de Conteúdo (IVC)^(29)^. Este método usa uma escala ordinal com quatro pontos, sendo o item mais baixo “discordo totalmente/não relevante” e o item mais alto “concordo totalmente/altamente relevante”^(29)^. O IVC é determinado pelo cálculo do número de itens cotados com 3 ou 4 a dividir pelo número total de itens^(29)^. Utilizou-se o valor de 0,78 como referência para assumir a existência de validade de conteúdo^(29)^.

Para validar o conteúdo do PIPA, relativamente à sua abrangência, clareza, adequação e pertinência dos conteúdos, constituíram-se dois painéis de peritos, com base nos critérios definidos na literatura^(25)^, designadamente, experiência clínica na área do PA (mínimo 5 anos) e conhecimento teórico na área de estudo. A seleção de dois painéis de peritos diferentes deve-se ao facto de se ter considerado pouco viável o mesmo PP avaliar um tão vasto conjunto de tarefas, associadas a um grande número de estímulos diferentes.

Os peritos foram selecionados de acordo com o método de amostragem não probabilístico por conveniência. O primeiro painel foi constituído por cinco peritos que analisaram as tarefas e o manual das competências auditivas de discriminação auditiva, atenção auditiva, memória auditiva e fechamento e o segundo painel por seis peritos que analisaram as competências de separação binaural, integração binaural, fusão binaural e figura-fundo.

Depois de um primeiro contacto por correio eletrónico, solicitando a colaboração dos peritos no estudo, foi enviado o manual do PIPA. Os peritos foram convidados a preencher um questionário dividido em duas partes: caracterização sociodemográfica e análise do conteúdo do PIPA (catorze afirmações, pontuadas de um a quatro)^(25,29)^. Com este questionário pretendeu-se atestar o cumprimento dos critérios de inclusão para integrar o PP e também avaliar a opinião dos especialistas relativamente aos seguintes itens: utilidade do programa, adequação à prática clínica e ao público-alvo, competências auditivas selecionadas, instruções, enquadramento, recompensas, tarefas, estímulos (quantidade e seleção) e organização.

## Resultados

A caracterização sociodemográfica dos peritos que analisaram o PIPA está descrita na Tabela 2.

**Tabela 2:** Constituição do PP

**Identificação dos Sujeitos**		**Género**	**Grau Académico**	**Anos de Profissão**	**Intervenção** *	Formação*	Formador*
**PP1**	Sujeito 1	Feminino	Licenciatura	5	Sim	Sim	Não
	Sujeito 2	Feminino	Licenciatura	27	Sim	Sim	Não
	Sujeito 3	Feminino	Doutoramento	17	Não	Sim	Não
	Sujeito 4	Feminino	Doutoramento	17	Sim	Sim	Sim
	Sujeito 5	Feminino	Licenciatura	20	Sim	Sim	Não
**PP2**	Sujeito 1	Feminino	Licenciatura	14	Sim	Sim	Sim
	Sujeito 2	Feminino	Licenciatura	20	Sim	Sim	Sim
	Sujeito 3	Feminino	Licenciatura	19	Sim	Sim	Sim
	Sujeito 4	Feminino	Mestrado	8	Sim	Sim	Não
	Sujeito 5	Feminino	Mestrado	14	Sim	Sim	Não
	Sujeito 6	Feminino	Doutoramento	12	Sim	Sim	Sim

* No âmbito do PA

Todos os peritos cumpriram os critérios pré-definidos de experiência clínica e de conhecimento específico na área do PA, destacando-se o facto de cinco dos onze peritos já terem ministrado formação em PA. Um dos peritos (Sujeito 3 – PP 1), à data do estudo, exercia funções de docência, mas conta também com experiência prévia ao nível da intervenção em indivíduos com PPA.

O IVC global obtido com a validação do PIPA é de 0,95. Os resultados quantitativos obtidos para o PP 1 e o PP 2 são apresentados na Tabela 3 e Tabela 4, respetivamente.

**Tabela 3:** Concordância obtida entre os elementos que integram o PP1, em relação ao PIPA

**Itens a validar/validados**	**IVC**
1. Estes materiais são úteis na prática clínica.	1
2. As competências auditivas selecionadas são adequadas.	1
3. As instruções apresentam clareza e pertinência prática.	0,8
4. O programa é adequado para crianças de idade escolar, com Perturbação do Processamento Auditivo.	1
5. O enquadramento do programa (uma menina que visita um jardim zoológico) é adequado.	1
6. A divisão por competências em diferentes espaços a visitar no jardim zoológico é adequada.	1
7. O *feedback*/recompensa dado em cada atividade é adequado.	1
8. As tarefas incluídas na secção *discriminação auditiva (baía dos golfinhos)* permitem a intervenção adequada com crianças com dificuldades nesta competência.	1
9. As tarefas incluídas na secção *atenção auditiva (alimentação dos pelicanos)* permitem a intervenção adequada com crianças com dificuldades nesta competência.	1
10. As tarefas incluídas na secção *memória auditiva (selva encantada)* permitem a intervenção adequada com crianças com dificuldades nesta competência.	1
11. As tarefas incluídas na secção *fechamento (parque pré-histórico)* permitem a intervenção adequada com crianças com dificuldades nesta competência.	0,6
12. Os estímulos selecionados para cada uma das tarefas são adequados.	0,8
13. O número de estímulos incluídos nas tarefas é adequado.	1
14. Dentro de cada competência, a organização das tarefas em níveis de dificuldade é adequada.	1
**Total global do PP1**	**0,94**

**Tabela 4:** Concordância obtida entre os elementos que integram o PP2, em relação ao PIPA

**Itens a validar/validados**	**IVC**
15. Estes materiais são úteis na prática clínica.	1
16. As competências auditivas selecionadas são adequadas.	1
17. As instruções apresentam clareza e pertinência prática.	0,83
18. O programa é adequado para crianças de idade escolar, com Perturbação do Processamento Auditivo.	1
19. O enquadramento do programa (uma menina que visita um jardim zoológico) é adequado.	0,83
20. A divisão por competências em diferentes espaços a visitar no jardim zoológico é adequada.	1
21. O *feedback*/recompensa dado em cada atividade é adequado.	1
22. As tarefas incluídas na secção *separação binaural (céu colorido)* permitem a intervenção adequada com crianças com dificuldades nesta competência.	1
23. As tarefas incluídas na secção *integração binaural (esconderijo dos rastejantes)* permitem a intervenção adequada com crianças com dificuldades nesta competência.	0,83
24. As tarefas incluídas na secção *fusão binaural (a quinta do tio Manel)* permitem a intervenção adequada com crianças com dificuldades nesta competência.	1
25. As tarefas incluídas na secção *figura-fundo (floresta mágica)* permitem a intervenção adequada com crianças com dificuldades nesta competência.	1
26. Os estímulos selecionados para cada uma das tarefas são adequados.	1
27. O número de estímulos incluídos nas tarefas é adequado.	1
28. Dentro de cada competência, a organização das tarefas em níveis de dificuldade é adequada.	1
**Total global do PP2**	**0,96**

Apesar de não ter sido necessária a completa reformulação de qualquer item, já que foi validado o conteúdo de todos os itens, foram realizadas algumas modificações ao PIPA, para atender às sugestões dos especialistas, registadas no campo destinado às observações/sugestões.

Relativamente ao enquadramento do programa, por sugestão dos peritos, foi introduzida a possibilidade de a criança escolher o género (masculino/feminino) da personagem principal do PIPA.

Foram realizadas alterações nas instruções das tarefas dos espaços C*éu colorido, Esconderijo dos rastejantes, A quinta do tio Manel* e *Floresta mágica*, tendo em vista uma melhor compreensão das mesmas por parte do TF e da criança, tendo sido utilizadas frases mais curtas e fornecidos exemplos. Em todas as atividades, foram introduzidos itens de treino, por sugestão dos peritos, no sentido de facilitar a compreensão da tarefa.

Para além disso, alteraram-se três pares de estímulos na tarefa *O salto do golfinho*, do espaço *Baía dos golfinhos,* de forma a aumentar a percentagem de palavras dissilábicas com o fonema em posição medial de palavra. Aumentou-se o número de estímulos na tarefa *O beijinho do cavalo marinho,* no espaço *Baía dos golfinhos,* passando de cinco para dez pares de pseudopalavras, de acordo com a recomendação dos peritos. Ainda no que reporta aos estímulos, por sugestão dos peritos, foram alteradas algumas frases, nas atividades *O voo da águia*, do espaço *Céu colorido* (separação binaural), e *Quantas cores tem o camaleão?*, do espaço *Esconderijo dos rastejantes* (integração binaural) tendo em vista a uniformização da sua estrutura gramatical.

Foi ainda acolhida a sugestão de não inserir no mesmo grupo de estímulos frases com agramaticalidade semântica e frases com agramaticalidade sintática, na tarefa *Quantas minhocas come a salamandra?,* do espaço *Esconderijo dos rastejantes* (integração binaural), optando-se apenas pela utilização de frases com agramaticalidade semântica. Foi também revisto o léxico de algumas frases, que por estar associado a um dialeto particular, poderia não ser familiar para crianças de outros pontos geográficos.

Nas tarefas de separação binaural (*Céu colorido*) foi contemplada a introdução de um maior desnível de intensidade (20 dB, 15 dB, 10dB, 5dB e sem desnível) entre os estímulos que surgem no lado direito *vs*. esquerdo, assim como foi tida em conta a sugestão de introduzir a possibilidade de manipular o desnível de intensidade (20 dB, 15 dB, 10dB, 5dB e sem desnível) nas tarefas de figura-fundo (*Floresta-mágica*), para ser garantida a usabilidade do PIPA nos casos de crianças com maior severidade de perturbação e/ou com perda auditiva associada.

A tarefa de memória auditiva de cores (nível 1 – *O corno do Rinoceronte*, no espaço *Selva Encantada*) foi igualmente reformulada, atendendo a que um dos peritos chamou a atenção para a questão do daltonismo. Nestas situações, a tarefa poderá ser realizada com o apoio do TF, que poderá selecionar as cores, depois da criança indicar a sequência de cores que ouviu.

No caso do item 11 do PP1, não houve a necessidade de alteração, mesmo tendo apresentado um IVC de 0.6, uma vez que, aquando do preenchimento do questionário, alguns elementos do PP não compreenderam que se tratava de uma tarefa na qual era utilizado ruído branco e/ou distorção.

Estas alterações encontram-se sumariadas na Tabela 5.

**Tabela 5:** Modificações realizadas no PIPA, por sugestão dos peritos

**Espaço**	**Alteração efetuada**	
Enquadramento do programa	Escolha do género (masculino/feminino) da personagem principal do PIPA	
Céu colorido	Alterações nas instruções	Introdução de um desnível de intensidade maior (20dB, 15dB, 10dB, 5dB e sem desnível) entre os estímulos
Esconderijo dos rastejantes		
A quinta do tio Manel		
Floresta mágica		Manipulação do desnível de intensidade (20dB, 15dB, 10dB, 5dB e sem desnível)
O salto do golfinho	Alteração de três pares de estímulos	
O beijinho do cavalo marinho	Aumento do número de estímulos	
O voo da águia	Alteração de algumas frases	
Quantas cores tem o camaleão?		
Quantas minhocas come a salamandra?	Manutenção apenas das frases com agramaticalidade semântica Revisão de todo o léxico	
O corno do rinoceronte	Reformulação da tarefa para permitir a sua realização por parte de crianças portadoras de daltonismo.	
Todas as atividades	Introdução de itens de treino	

## Discussão

A seleção de dois painéis de peritos, um com cinco elementos e outro com seis, revelou-se adequada, já que se encontra dentro do intervalo de cinco a dez peritos mencionado na literatura^(25)^. O facto de os peritos terem experiência clínica e formação na área do PA demonstra que o avaliador está familiarizado com esta área, o que justifica, desde logo, a sua inclusão no PP.

O facto de os elementos de cada PP não conhecerem o PIPA no seu todo terá dificultado o trabalho de análise solicitado, apesar disso, cumpriram-se os requisitos de um estudo de validação de conteúdo, selecionando especialistas de várias zonas geográficas do território nacional continental que cumprem todos os critérios de inclusão^(25)^.

O IVC global obtido (0,95) corresponde a uma validade de conteúdo excelente, uma vez que é superior a 0,90^(25,29,30)^. Contudo, foram realizadas alterações, uma vez que os peritos se encontravam de acordo em relação às sugestões^(29)^. Caso contrário, Polit & Beck^(29)^ defendem que deverá ser novamente revista a literatura para melhorar o programa.

O desenvolvimento e validação de um programa de intervenção, particularmente no que reporta à área do PA, constitui um fator inovador para o PE. Neste âmbito, e dada a escassez de materiais, optou-se por criar um programa em que são utilizados estímulos verbais para o treino das diferentes competências auditivas^(10,17)^. Neste sentido, foi fundamental a seleção cuidada dos estímulos verbais que foram incluídos no PIPA, balanceados de acordo com os padrões de frequência do PE^(27,28)^.

Para a elaboração do PIPA procurou realizar-se uma seleção cuidada de competências, objetivos, tarefas e estímulos, tendo sempre em vista a sua utilização em contexto clínico. Paralelamente, foi tido em consideração o facto de o programa ter como público-alvo crianças em idade escolar e, como tal, ser necessária a utilização de ferramentas lúdicas como forma de motivação e adesão às mesmas^(8,9)^.

A inclusão de um sistema de recompensas e de monitorização de acertos por parte da criança, para além de ter sido validada por unanimidade pelos peritos, constitui um fator fundamental na avaliação contínua da criança, na (re)definição de objetivos de intervenção e na manutenção de índices motivacionais para a tarefa, estando de acordo com o que é defendido na literatura para a intervenção em casos de PPA^(9,18)^. Para além disso, está em conformidade com alguns programas disponíveis internacionalmente, que mostram os progressos da criança nas atividades de treino auditivo^(20,22)^.

As competências auditivas desenvolvidas no PIPA (discriminação auditiva, atenção auditiva, memória auditiva, separação binaural, integração binaural, fusão binaural, fechamento e figura-fundo) permitem a compreensão de fala, uma vez que envolvem a discriminação, o reconhecimento, a atenção seletiva e sustentada dos sons e a capacidade de memorização dos mesmos^(11)^. Desta forma, e atendendo a que a PPA pode ter consequências negativas para o desempenho linguístico, social e académico dos indivíduos^(7,11)^, o PIPA poderá ter um impacto positivo nos seus utilizadores, no que concerne aos fatores pessoais que promovem a atividade e participação nos vários contextos.

Para além disso, a prática baseada na evidência na tomada de decisões é fundamental para elevar a qualidade da intervenção terapêutica^(7)^. O PIPA abre assim caminho para outros estudos no âmbito da intervenção na PPA em crianças cuja língua materna é o PE, contribuindo para melhoria da prática clínica dos TFs nesta área. Como trabalho futuro, estão previstos estudos de aceitabilidade e eficácia do PIPA, junto de crianças com e sem PPA.

## CONCLUSÃO

Este trabalho permitiu o desenvolvimento e validação do PIPA, cumprindo com as etapas definidas na literatura para a criação de novos instrumentos. Trata-se de um instrumento inovador para o PE, com um IVC excelente, cuja análise da sua aceitabilidade e eficácia está prevista, como trabalho futuro.
